# A comprehensive review of the genetics of juvenile idiopathic arthritis

**DOI:** 10.1186/1546-0096-6-11

**Published:** 2008-07-21

**Authors:** Sampath Prahalad, David N Glass

**Affiliations:** 1 Assistant Professor of Pediatrics, Division of Immunology and Rheumatology, Department of Pediatrics, University of Utah School of Medicine, P.O Box 581289 Salt Lake City, UT 84158-1289, USA; 2 Professor of Pediatrics, University of Cincinnati College of Medicine, Cincinnati Children's Hospital Medical Center, 3333 Burnet Ave., MLC 7030, Cincinnati, OH 45229, USA

## Abstract

Juvenile idiopathic arthritis (JIA) is the most common chronic arthropathy of childhood which is believed to be influenced by both genetic and environmental factors. The progress in identifying genes underlying JIA susceptibility using candidate gene association studies has been slow. Several associations between JIA and variants in the genes encoding the human leukocyte antigens (HLA) have been confirmed and replicated in independent cohorts. However it is clear that genetic variants outside the HLA also influence susceptibility to JIA. While a large number of non-HLA candidate genes have been tested for associations, only a handful of reported associations such as *PTPN22 *have been validated. In this review we discuss the principles behind genetic studies of complex traits like JIA, and comprehensively catalogue non-*HLA *candidate-gene association studies performed in JIA to date and review several validated associations. Most candidate gene studies are underpowered and do not detect associations, and those that do are often not replicated. We also discuss the principles behind genome-wide association studies and discuss possible implications for identifying genes underlying JIA. Finally we discuss several genetic variants underlying multiple clinically distinct autoimmune phenotypes.

## 

Juvenile idiopathic arthritis rers to a group of chronic arthropathies of childhood[1]. According to the International League of Associations for Rheumatology criteria, JIA comprises seven subtypes[2]. These include systemic JIA (sJIA), oligoarticular JIA, rheumatoid factor (RF)-negative polyarticular JIA, RF-positive polyarticular JIA, enthesitis related arthritis (ERA), psoriatic arthritis and undifferentiated JIA. The relatively homogeneous subtypes of JIA share clinical features with other chronic autoimmune disorders such as rheumatoid arthritis (RA), psoriasis or spondyloarthropathies [3]. Autoimmune disorders are relatively common in the population, estimated to have a prevalence of about ~5% in the US. While clinical and laboratory features distinguish many of these autoimmune disorders, there is accumulating evidence to support the hypothesis that clinically distinct autoimmune phenotypes share common genetic susceptibility factors. In this review, we will examine the genetic factors that underlie JIA susceptibility, and discuss some of the genetic factors that underlie multiple autoimmune phenotypes.

## JIA is an autoimmune disorder

All subtypes of JIA are characterized by persistent joint swelling caused by an accumulation of synovial fluid and thickening of the synovial lining. There is evidence to support the involvement of different components of the immune system in the etiopathogenesis of JIA. The synovial tissue contains various inflammatory cells including neutrophils, plasma cells, dendritic cells and a high proportion of activated T-cells [4-6]. The recruitment of pro-inflammatory cells into the synovium of a child with JIA is believed to be mediated by chemokines that selectively attract Th1 T-cells [7-9]. These T-cells are characterized by the production of interleukin (IL)-2, interferon-γ, and tumor necrosis factor (TNF)-β. Many other autoimmune disorders including RA, inflammatory bowel disease (IBD), psoriasis, and type 1 diabetes mellitus (T1DM) are also associated with Th1-dominant responses[10,11]. Several studies have demonstrated that Th1 cytokines also predominate in the synovial tissue and synovial fluid samples from children with JIA [12-15]. Pro-inflammatory cytokines including sCD154 are significantly elevated in sera of children with JIA as well [16]. Thus, there is compelling evidence that activated T-lymphocytes play a role in the pathogenesis of JIA.

Involvement of the other components of the immune system is also evident in JIA. For instance a significant proportion of children with JIA have antinuclear antibodies, and some children have rheumatoid factors, suggesting a role of the humoral component of the immune system. The predominance of neutrophils in the synovial fluid of children with JIA, elevated levels of monocyte derived inflammatory cytokines, and complement activation have led to investigations of the involvement of innate immune system in JIA. Using gene expression analysis, Jarvis et al have found that neutrophils in children with polyarticular JIA show differences in levels of expression of over 700 genes compared to healthy controls [17]. These differences persisted despite clinical responses to pharmacological therapy, suggesting that neutrophils might be intrinsically involved in the pathogenesis of polyarticular subtypes of JIA. Together, these data point to the role of aberrant immune/inflammatory responses in JIA.

## Evidence that genetic factors underlie JIA susceptibility

Twin and family studies have provided evidence for genetic contributions to susceptibility of JIA. Twin studies have shown that the monozygotic twin concordance rates for JIA range between 25 to 40 %, a risk that is substantially greater than the population prevalence of 1 in 1000 [18]. An analysis of twin pairs in a national JIA registry showed that at least 80 % of the twin pairs in the registry were monozygotic, when one would expect only a third to be monozygotic [19]. Similarly examination of affected sibling pairs with JIA reveals concordance for disease onset, and disease course[20,21]. Furthermore siblings were more likely to develop JIA at the same age of onset, rather than the same calendar year. The prevalence of JIA among siblings of probands is about 15–30 times that of the general population prevalence[22,23]. Finally a computerized probabilistic record-linking analysis identified several clusters of children with JIA sharing common ancestors [23]. Together these studies provide compelling evidence for a substantial genetic component underlying JIA susceptibility.

## JIA is a complex genetic trait

Complex genetic traits are those phenotypes that do not exhibit classic Mendelian inheritance patterns which could be attributed to variants in a single gene locus[24,25]. Complex traits are often believed to be determined by a number of genetic and environmental factors. While increased prevalence of JIA has been shown in twins or siblings of probands with JIA, parent-offspring pairs and extended multiplex JIA families are relatively uncommon in JIA. Relatives of JIA probands have increased prevalence of other autoimmune disorders [26,27]. The best characterized genetic factors, i.e., polymorphisms in the genes encoding human leukocyte antigen (HLA -DR) have been estimated to account for only 17 % of the proportion of sibling recurrence risk in JIA[28]. These factors support the notion that JIA is a complex phenotype[22].

## Exploring the genetic basis of disease

Linkage and association studies are analytical approaches used to dissect the genetic basis of common diseases. Both studies rely on the same principles and assumptions, namely the co-inheritance of polymorphisms linked to a disease allele [29,30]. Linkage studies test whether a phenotype and a marker allele show correlated transmission within a pedigree[24]. Linkage analysis requires large collections of multiplex families, in which multiple family members have the phenotype of interest. Association studies test whether a phenotype and a marker allele show correlated occurrence in a population[24]. Association studies compare unrelated affected and unaffected individuals within a population. An allele at a locus of interest is associated with a phenotype if it occurs at a significantly higher (or lower) frequency among cases compared to controls. The selection of controls is very critical in case-control association studies, since results can be affected by population stratification. Family-based association studies, including the transmission disequilibrium test (TDT) are not affected by population stratification, since they use untransmitted alleles as controls within families. When linkage or association between a marker and a disease is detected, it implies either that the marker is the disease allele, or that the marker is in linkage disequilibrium (LD) with the disease allele. LD is a non-random association of alleles at two or more loci and is a measure of co-segregation of alleles in a population[31,32].

The search for genetic factors underlying susceptibility to complex traits can be classified as functional candidate gene studies or genome-wide studies. Functional candidate-gene studies begin by selecting genes whose known functions suggest that they could be influencing the phenotype of interest. In genome-wide studies, the genome is searched for susceptibility loci, and no assumptions are made about the candidacy of particular genes or genomic regions. Until now most association studies could examine only a small fraction of the sequence variation in an affected individual. However, advances in genome technologies, and the availability of haplotype and LD information from the HapMap project now facilitate comprehensive genome-wide searches for genetic influences [29].

## Genes underlying JIA susceptibility

Genetic variants underlying JIA susceptibility have been reviewed extensively[18,33-37]. The major histocompatibility complex (MHC) region on chromosome 6 is packed with over 200 genes, many of which are essential to the immune system. Numerous associations between HLA polymorphisms and JIA subtypes have been reported in multiple populations (Table 1). Within the HLA complex there are genes encoding class I (HLA A, B and C), and class II (HLA-DR, DP and DQ) molecules. The class I allele *HLA-A2 *is associated with different JIA subtypes, especially in those with early onset[38,39]. *HLA-B27 *is associated with enthesitis related JIA [39-41]. Oligoarticular JIA is the subtype with the most HLA class II associations. Oligoarticular JIA is positively associated with HLA DRB1*01, DRB1*08, DRB1*11, DRB1*13, DPB1*02 and DQB1*04[38,39,41-44]. HLA DRB1*04 and DRB1*07 are seen less frequently in children with oligoarticular JIA than controls, suggesting that they are protective[38,39,41,44]. Polyarticular RF negative JIA is associated with DRB1*08 and DPB1*03 [41,44]. Polyarticular RF-positive JIA, which is phenotypically similar to adult RA, is associated with DRB1*04, DQA1*03, and DQB1*03[41,44]. Psoriatic arthritis was found in the study by Thomson et al to be associated with HLA DRB1*01, and DQA1*0101[41]. Associations between sJIA and HLA DRB1*04 have also been reported[45,46]. A recent study reported an association between a polymorphism in the HLA-G region and JIA, with a pronounced association in female subjects [47]. While some associations appear to be secondary to LD (for instance HLA DR and DQ alleles), most associations are not explained by LD, suggesting that several HLA loci independently contribute to susceptibility of JIA or its subtypes. A study involving over 600 children with JIA and 254 ethnicity-matched healthy controls confirmed several previously reported HLA associations, and also identified age-specific windows of susceptibility for the different HLA alleles[39]. This study also demonstrated that the number of susceptibility alleles was inversely proportional to the age of onset of JIA. Among children who carried HLA-A2, DPB1*0201, and one susceptible -DR allele the median age of onset of oligoarticular JIA was just 2.4 years.

**Table 1:** Associations between JIA subtypes and different HLA alleles.

Phenotype	Alleles conferring susceptibility	Protective alleles
Oligoarticular	A2, DRB1*01, DRB1*08, DRB1*11, DRB1*13, DPB1*02, DQA1*04, DQB1*04	DRB1*04, DRB1*07, DQA1*03
Polyarticular RF+	DRB1*04, DQA1*03, DQB1*03	DQA1*02
Polyarticular RF-	A2, DRB1*08, DQA1*04, DPB1*03	
Psoriatic	DRB1*01, DQA1*0101	DRB1*04, DQA1*03
ERA	B27, DRB1*01, DQA1*0101, DQB1*05	
Systemic	DRB1*04, DRB1*11, DQA1*05	

## Non HLA Associations

A number of non-HLA genes have been tested for association with JIA. Only a few of these have been replicated in additional cohorts. Some of these are reviewed by Phelan et al [33]. A systematic review of the literature suggests that about 100 different non-HLA candidate loci have been investigated for associations with JIA, in over 150 tests for genetic association in different cohorts (Table 2). Although ~25 % of all tests reported finding an association, independent confirmations are found for only a handful of candidate genes including *PTPN22, MIF, SLC11A6, WISP3*, and *TNFA *(Table 3). Many studies analyze JIA as a single phenotype. While combining clinically distinct entities such as systemic JIA and oligoarticular JIA might confound the results, stratification of subjects leads to further loss of power. Finally most studies do not correct for multiple comparisons, which might result in false positive associations that cannot be ultimately confirmed. The ranges of cases in these studies varied from 33 individuals to 950 individuals with JIA, and the number of controls ranged from 40 to 1952. The median number of cases was 130, while the median number of controls was 276. Only a few studies used more than 800 cases of JIA. These observations suggest that most of these studies are underpowered to detect associations with modest odds ratios (OR) (Table 4). Results of studies in other complex genetic traits suggest that most associated variants have only a modest OR of about 1.5. These observations reinforce the importance of properly performed and reported studies including the capacity to replicate.

**Table 2:** Non-HLA candidate markers tested for association with JIA.

**Marker**	**ASSN**	**Cases**	**Controls**	**Design**	**NOTES**	**Author**	**ID**
ACE	**Y**	82	48	CC	Insertion/deletion polymorphism	Alsaid	[119]
ADAM33	N	86	270	CC		Schubert	[120]
ADRB2	N	86	270	CC		Schubert	[120]
ADRB2	N	348	448	CC		Pont-Kingdon	[121]
Alpha 1 AT	**Y**	96	4565	CC		Arnaud	[122]
BV13S2	N	120	500	CC		Epplen	[56]
BV62&	N	120	500	CC		Epplen	[56]
BV6S3	N	120	500	CC		Epplen	[56]
CARD15	N	86	270	CC		Schubert	[120]
CBG	N	463	276	CC		Donn	[123]
CCR3	N	86	270	CC		Schubert	[120]
CCR5	N	86	270	CC		Schubert	[120]
CCR5	**Y**	819		TDT	Protective	Prahalad	[79]
CCR5	N	524	658	CC		Lindner	[80]
CCR5	**Y**	101	104	CC	Positive association in systemic+poly	Scheibel	[124]
CD3D	N	120	500	CC		Epplen	[56]
CD40L	N	120	500	CC		Epplen	[56]
CRH	N	463	276	CC		Donn	[123]
CSF2	N	86	270	CC		Schubert	[120]
CTLA4	**Y**	197	362	CC		Miterski	[59]
CTLA4	N	72	475	CC		Suppiah	[125]
CTLA4	N	86	270	CC		Schubert	[120]
CTLA4	N	818	518	CC		Prahalad	[113]
CTSL2	N	530	559	CC		Viken 2007	[126]
CYP19	N	463	276	CC		Donn	[123]
D17S795	N	173	416	CC		Miterski	[59]
D17S807	N	172	412	CC		Miterski	[59]
D17S821	N	173	412	CC		Miterski	[59]
DDR1	N	135	199	TDT		Zeggini	[127]
ESR1	N	463	276	CC		Donn	[123]
FAS	N	342	255	CC		Donn	[128]
FCER1B	N	86	270	CC		Schubert	[120]
FCRL3	**Y**	524	1030	CC	Association seen in poly JIA subset	Eike	[129]
FGFA	N	120	500	CC		Epplen	[56]
FOXp3	N	761	402	CC		Eastell	[130]
GSTM1	N	103	90	CC		Rohr	[131]
GSTP1	N	103	90	CC		Rohr	[131]
GSTT1	**Y**	103	90	CC		Rohr	[131]
GZMB	N	133	384	CC	Only systemic JIA studied	Donn	[95]
IFNA	N	120	500	CC		Epplen	[56]
IFNA1	N	417	276	CC		Donn	[69]
IFNG	N	417	276	CC		Donn	[69]
IFNG	N	165	395	CC		Miterski	[59]
IFNG	N	130	103	CC		Cinek	[132]
IgA def	**Y**	1673		CC	Data derived from several studies.	Cassidy	[133]
IKBL	N	170	389	CC		Miterski	[59]
IL1 cluster	**Y**	235	335	CC	Only systemic JIA studied. Two stage study	Stock	[71]
IL1R cluster	**Y**	235	335	CC	Only systemic JIA studied. Two stage study	Stock	[71]
IL1A	N	417	276	CC		Donn	[69]
IL1A	**Y**	269	99	CC	Association with early onset oligo JIA	McDowell	[68]
IL1A	N	130	103	CC		Cinek	[132]
IL1A	N	120	500	CC		Epplen	[56]
IL1B	**Y**	107	630	CC		Cimaz	[134]
IL1B	N	130	103	CC	Non-significant association reported.	Cinek	[132]
IL1R	N	130	103	CC		Cinek	[132]
IL1RA	N	107	630	CC		Cimaz	[134]
IL1RN	**Y**	235	306	CC		Vencovsky	[135]
IL2	N	417	276	CC		Donn	[69]
IL2	N	86	270	CC		Schubert	[120]
IL2	N	130	103	CC		Cinek	[132]
IL2	N	120	500	CC		Epplen	[56]
IL3	N	86	270	CC		Schubert	[120]
IL4	N	417	276	CC		Donn	[69]
IL4	N	86	270	CC		Schubert	[120]
IL4	N	72	165	CC	Trend in female cases	Suppiah	[136]
IL4	**Y**	130	103	CC		Cinek	[132]
IL4R	N	72	165	CC	Trend in female cases	Suppiah	[136]
IL4RA	N	130	103	CC		Cinek	[132]
IL5RA	N	120	500	CC		Epplen	[56]
IL6	N	417	276	CC		Donn	[69]
IL6	**Y**	222		TDT	Only systemic JIA patients studied	Ogilvie	[76]
IL6	N	86	270	CC		Schubert	[120]
IL6	N	130	103	CC		Cinek	[132]
IL8	N	86	270	CC		Schubert	[120]
IL10	N	417	276	CC		Donn	[69]
IL10	N	86	270	CC		Schubert	[120]
IL10	**Y**	172	473	CC	Only systemic JIA patients studied	Fife	[137]
IL10	N	130	103	CC		Cinek	[132]
IL12	N	130	103	CC		Cinek	[132]
IL13	N	86	270	CC		Schubert	[120]
IL15	**Y**	107	270	CC		Bierbaum	[138]
IL18	N	86	270	CC		Schubert	[120]
IL18	**Y**	33	176	CC		sugiura	[139]
IL1A	N	417	276	CC		Donn	[69]
IL1A	**Y**	269	99	CC	Association with early onset oligo JIA	McDowell	[68]
IL1A	N	130	103	CC		Cinek	[132]
IL1A	N	120	500	CC		Epplen	[56]
IL1B	**Y**	107	630	CC		Cimaz	[134]
IL1B	N	130	103	CC	Non-significant association reported.	Cinek	[132]
IL1R	N	130	103	CC		Cinek	[132]
IL1RA	N	107	630	CC		Cimaz	[134]
IL1RN	**Y**	235	306	CC		Vencovsky	[135]
IL2	N	417	276	CC		Donn	[69]
IL2	N	86	270	CC		Schubert	[120]
IL2	N	130	103	CC		Cinek	[132]
IL2	N	120	500	CC		Epplen	[56]
IL3	N	86	270	CC		Schubert	[120]
IL4	N	417	276	CC		Donn	[69]
IL4	N	86	270	CC		Schubert	[120]
IL4	N	72	165	CC	Trend in female cases	Suppiah	[136]
IL4	**Y**	130	103	CC		Cinek	[132]
IL4R	N	72	165	CC	Trend in female cases	Suppiah	[136]
IL4RA	N	130	103	CC		Cinek	[132]
IL5RA	N	120	500	CC		Epplen	[56]
IL6	N	417	276	CC		Donn	[69]
IL6	**Y**	222		TDT	Only systemic JIA patients studied	Ogilvie	[76]
IL6	N	86	270	CC		Schubert	[120]
IL6	N	130	103	CC		Cinek	[132]
IL8	N	86	270	CC		Schubert	[120]
IRF1	**Y**	417	276	CC		Donn	[69]
IRF1	N	765	508	CC		Fife	[140]
IRF2	N	120	500	CC		Epplen	[56]
LMP 7	**Y**	207	50	CC		Prahalad	[141]
MBL	N	93	48	CC		Kang	[142]
MCP1	N	86	270	CC		Schubert	[120]
MCP1	N	66	150	CC		Ozyurek	[143]
MEFV	**Y**	71	100	CC	Included 12 subjects with vasculitis	Ozen	[144]
MEFV	**Y**	950	728	CC	Association in psoriatic JIA after correction for multiple testing	Day	[145]
MHC2TA	**Y**	74	316	CC		O'Doherty	[146]
MICA	**Y**	128	113	CC	Association with A4 allele	Nikitina Zake	[147]
MIF	**Y**	526	259	CC		Donn	[60]
MIF	N	150	390	CC		Miterski	[59]
MIF	**Y**	224	341	CC	Association with allele 05	Miterski	[59]
MIF	**Y**	321		TDT		Donn	[61]
MIF	N	86	270	CC		Schubert	[120]
MIF	N	67	153	CC		Berdeli	[62]
MTHFR	N	56	62	CC		Huemer	[148]
MTR	N	56	62	CC		Huemer	[148]
MTRR	N	56	62	CC		Huemer	[148]
NLP3	**Y**	950	728	CC	Association in psoriatic JIA after correction for multiple testing	Day	[145]
NOD2	N	950	728	CC	Association in psoriatic JIA ***before ***correction for multiple testing	Day	[145]
NOS33	N	86	270	CC		Schubert	[120]
Osteopontin	N	119	200	CC	Oligo JIA only.	Marciano	[149]
PAFAH	N	86	270	CC		Schubert	[120]
PRF1	N	133	384	CC	Only systemic JIA studied	Donn	[95]
PRL	N	463	276	CC		Donn	[123]
PSTPIP1	N	950	728	CC	Association in psoriatic JIA before correction for multiple testing	Day	[145]
PTPN22	**Y**	661	595	CC		Hinks	[84]
PTPN22	N	230	1400	CC		Seldin	[85]
PTPN22	**Y**	320	555	CC		Viken	[86]
PTPN22	**Y**	130	400	CC		Cinek	[87]
PTPRC	N	161	362	CC		Miterski	[59]
Rab27a	N	133	384	CC	Only systemic studied	Donn	[95]
RANTES	N	86	270	CC		Schubert	[120]
SELP	N	86	270	CC		Schubert	[120]
SH2D2A	**Y**	210	558	CC		Smerdel	[150]
SLC11A1	**Y**	119	111	CC	Also named NRAMP1	Sanjeevi	[66]
SLC11A1	**Y**	234	639	TDT		Runstadler	[67]
SLC26A2	**Y**	826	617	CC	Association seen only in systemic jia	Lamb	[151]
SUMO4	N	668	484	CC		Gibbons	[152]
TAP	**Y**	285	165	CC	Despite correcting for HLA	Ploski	[153]
Tapasin	**Y**	156		TDT	Only systemic studied	Bukulmez	[154]
TCRBv6.1	**Y**	126	207	CC	Association in HLADQA1*0101 positive subjects	Maksymowych	[155]
TCRBv6.1	N	77	40	CC	only HLADQA1*0101 positive subjects	Ploski	[156]
TCRBv6.1	N	120	500	CC		Epplen	[56]
TCRDVAJ	N	120	500	CC		Epplen	[56]
TEA	N	120	500	CC		Epplen	[56]
TGFB1	N	130	103	CC		Cinek	[132]
TLR4	N	313		TDT		Lamb	[157]
TLR4	N	86	270	CC		Schubert	[120]
TLR9	N	86	270	CC		Schubert	[120]
TNFA	**Y**	128	114	CC	Association with A2 allele	Nikitina Zake	[147]
TNFA	**Y**	142	388	CC	Association with allele 6	Miterski	[59]
TNFA	N	170	415	CC		Miterski	[59]
TNFA	**Y**	228	196	CC	Association in psoriatic, RF-negative subtypes	Schmeling	[58]
TNFA	N	86	270	CC		Schubert	[120]
TNFA	N	55	68	CC	Difference between oligo and systemic	Modesto	[158]
TNFA	N	107	630	CC		Cimaz	[134]
TNFA	**Y**	120	500	CC	Microsatellite	Epplen	[56]
TNFB	N	128	114	CC		Nikitina Zake	[147]
TNFB	N	86	270	CC		Schubert	[120]
TNFR1	N	132	334	CC		Miterski	[59]
TNFR2	N	435	261	CC		Zeggini	[159]
TNFR2	N	146	428	CC		Miterski	[59]
TRAF1	**Y**	67	1952	CC	Subjects genotyped as part of genome-wide association	Behrens	[160]
UNC13D	N	133	384	CC	Only systemic JIA studied	Donn	[95]
WISP3	**Y**	159	263	CC		Lamb	[77]
WISP3	**Y**	181	355	CC	Replication study	Lamb	[77]

Marker: Gene or locus studied for association. Assn: Association; CC: case-control; TDT: transmission-disequilibrium testing.

**Table 3:** Non-HLA genetic genes associated with JIA that have been independently confirmed.

PTPN22
TNFA
MIF
WISP3
SLC11A1

**Table 4:** Sample sizes requirements for genetic association studies

MAF	10%		20%		30%		40%	
	Cases	controls	Cases	controls	Cases	Controls	Cases	Controls

OR								
2.0	223	446	137	274	113	226	107	214
1.5	703	1406	415	830	332	664	304	608
1.3	1751	3502	1016	2032	798	1596	719	1438

Table shows number of cases and controls that need to be typed to detect associations at a significance value of 0.05, power of 80%, for various minor allele frequencies, and odds ratios. A case: control ratio of 1:2 is assumed. OR: odds ratio; MAF: minor allele frequency.

Interpreting the different associations (or lack of associations) between genetic variants and phenotypes can be further complicated by differences in phenotype description. JIA comprises several sub-phenotypes with distinct clinical features and outcomes[48]. Although the ILAR classification criteria, which are increasingly being used to describe juvenile arthritis attempts to define homogenous subtypes, there are still some challenges. For instance a child with four affected joints is classified as oligoarticular JIA, while a child with five affected joints is classified as polyarticular JIA, although clinically the difference is not significant. Similarly, a child with five affected joints and another with over 20 joints are both classified as polyarticular, although clinically they appear distinct. While this can be addressed in part, by analyzing children with JIA as a group, and then performing analyses by subtypes, this results in multiple comparisons. One approach to address this issue might be to use genetic information to stratify subjects into subtypes, and this might results in more homogenous subtypes. For instance studies of adult RA subjects frequently stratify the subjects based on presence or absence of the shared *HLA-DR *epitope[49,50]. Similarly, it might be reasonable to stratify JIA patients by HLA associations while conducting genetic studies. It also would better facilitate comparisons of genetic studies performed in different populations. Finally, this would also help to delineate associations due to LD. For instance the MHC region has extensive LD, and associations described with variants in this region could be due to LD with HLA polymorphisms. Together, these observations reinforce the need for meticulous attention to defining the phenotypes and caution with interpreting results of genetic association studies.

A recent critical review of several genes and polymorphisms tested as part of commercially available genomic profiles highlights the challenges of genetic association testing [51]. The authors used individual gene-disease association studies as well as meta-analyses to examine polymorphisms in 56 genes. Of these 32 genes had been examined in meta-analyses of 160 polymorphism-disease association comparisons. Only 38% were found to be statistically significant, with very modest associations. Furthermore, often associations were found with phenotypes different from the original phenotype being tested for association. For instance, genes tested in cardiogenomic profiles were more frequently associated with non-cardiac diseases. It is anticipated that most disease-phenotype associations of complex traits are likely to be of modest significance, with OR <1.5 [52]. Thus, the modest genetic effects necessitates collaboration, both by performing collaborative studies where samples are pooled, as well as by data-sharing by investigators in order to detect meaningful associations[53].

Non-replication of initial associations can be due to myriad factors including a false-positive result in the first cohort, population stratification in the first cohort, inadequate power in the replication cohort(s), genotyping errors, selection biases, and true population differences. It should be noted that lack of an association in the first cohort often might discourage further studies of that gene by other investigators, even though the first cohort might not have had adequate power to truly detect a modest association. The power to detect associations also depends on the minor allele frequency, and hence for rare variants much larger samples would be necessary. It is feasible to increase the power of genetic studies by using large numbers of appropriate controls, thereby increasing the control to case ratio. The quality of genetic associations will be significantly enhanced by increasing sample sizes, and validating initial discoveries by collaborative studies. Following published guidelines for performing and reporting genetic association studies will improve the quality of studies and facilitate the discovery of true causal variants[54,55]. For instance, providing power calculations, and if the variant under study has been investigated previously, performance of a meta-analysis would be beneficial. Ultimately, associations that are reproducible, as well as demonstration of functional consequences of genetic variation are necessary to translate the results of these studies to the diagnosis, treatment and understanding of JIA.

Association studies between JIA and non-HLA variants have been reviewed previously [33-35]. The following are some genetic associations that have been confirmed in more than one cohort. *TNFA*, the gene encoding the pro-inflammatory cytokine TNF-α has been the focus of several association studies of autoimmune diseases. Associations between *TNFA *and different JIA subtypes have been previously reported. An association between early onset oligoarticular JIA and TNF variants were reported by Epplen et al[56]. Another study found associations between persistent oligoarticular JIA and *TNFA *variants[57]. An association between a SNP at position -857 of *TNFA *and sJIA was reported by Date et al[45]. Schmeling et al found an association between *TNFA *variants and psoriatic arthritis polyarticular JIA subtypes[58]. In a case control study of children with JIA, Miterski et al found no associations with *TNFA *SNPs, but found an association with a microsatellite in the *TNFA *gene[59]. Thus, while variants in TNFA appear to be associated with JIA, there are clearly differences between various studies with regard to the associated variant(s) and/or associated subtype. It should be noted that some of the associations could be due to LD, as the *TNFA *and *HLA *loci are both contained in the MHC region.

The gene encoding macrophage migration inhibitory factor (MIF) has also been investigated in several cohorts of patients with JIA. A functional SNP at position -173 has been associated with JIA in the study by Donn et al, in 526 British children with JIA and 259 ethnicity-matched controls[60]. This finding was replicated using an independent cohort of JIA trios, using TDT [61]. However, this SNP did not show an association in the studies by Miterski et al, and Berdeli et al, although it should be noted that both of these cohorts had smaller number of subjects [59,62]. Of interest, the latter study did find that carriage of -173C allele was a strong predictor of poor outcome of JIA[62]. It has previously been shown that this allele is a predictor of poor outcome in sJIA [63]. A recent study of patients with oligoarticular JIA that had been followed for at least 5 years confirmed that -173C was a predictor of poor response to intra-articular corticosteroid treatment [64]. Together, these studies imply that MIF variants may be associated with susceptibility to JIA, as well as the phenotype of JIA but should be noted that *MIF-173C *was not associated with adult RA in a replication study[65].

The *SLC11A1 *gene (formerly called *NRAMP1*) is important in natural resistance to various intracellular infections mediated by macrophages. Variants in this gene have also been found to be associated with JIA in at least two cohorts [66,67]. In the first study of Latvian and Russian children with JIA, a functional promoter microsatellite polymorphism (allele 3) was found to be associated with susceptibility to JIA (OR 2.26)[66]. Conversely, another variant, allele 2, was associated with protection from JIA. Interestingly, these alleles demonstrate opposite associations in infection and autoimmunity, with allele 3 conferring susceptibility to autoimmunity and protection from tuberculosis. Conversely, allele 2 confers protection from autoimmunity while increasing susceptibility to tuberculosis. These suggest that balancing natural selection might be acting upon this locus. In a subsequent study, Runstadler et al studied both of these *SLC11A1 *alleles as well as three SNPs and an insertion/deletion polymorphism in the region [67]. A six-marker haplotype demonstrated a strong association with JIA as a group, as well as in oligoarticular and polyarticular subtypes. Haplotypes that did not contain allele 3 identified in the study by Sanjeevi et al, were also found to show significant association suggesting that variants in this region might have independent associations with JIA. These two studies support a role for *SLC11A1 *variants in JIA susceptibility.

The gene encoding IL-1α, a potent pro-inflammatory cytokine, has been investigated in several JIA cohorts for a genetic association. Originally an association was described between a SNP in the promoter region of *IL1A *in Norwegian children with JIA[68]. The association was most pronounced in those with early onset oligoarticular JIA. However this finding was not replicated in two cohorts from the UK in subsequent studies, although one study investigating a number of polymorphisms demonstrated an association that was not significant when corrected for multiple comparisons [69,70]. A recent two-staged association study identified several variants in the IL-1 gene cluster, and some in the IL-1 receptor cluster were associated with systemic JIA [71]. IL-6 is another potently pro-inflammatory cytokine shown to be elevated in children with JIA[16,72-74]. A functional SNP in the promoter of the *IL6 *gene was shown to be associated with sJIA by Fishman et al. The effect was pronounced in children under 5 years of age[75]. This finding was not replicated in a smaller sJIA cohort by Donn et al[69]. However, using an international cohort of families of children with sJIA, Ogilvie et al did find a significant association between the IL6-174 SNP and sJIA, but in children with onset >5 years[76]. An association between a promoter variant in *IRF1*, the gene encoding interferon regulatory factor and JIA was reported by Donn et al in a cohort of 417 cases with JIA and 276 controls[69]. However, when they repeated the study using different controls and additional cases, no significant differences in allele or genotype frequencies of several *IRF1 *variants were observed. Together, these studies illustrate the difficulties of replicating significant positive findings from initial cohorts in subsequent larger replication studies.

An association between a SNP (G84A) in the gene encoding the Wnt-1 inducible signaling pathway protein 3 (*WISP3*) and polyarticular JIA was described by Lamb et al[77]. After observing a positive association in the initial cohort of 159 cases and 263 controls, the finding was replicated in an independent cohort of 181 cases and 355 controls. In addition they used parent-child trios for TDT, which demonstrated a trend towards over-transmission of the 84A allele. This finding has not been replicated in other cohorts to our knowledge.

JIA and RA are examples of autoimmune diseases mediated by Th1-immune responses. Synovial T-cells express high levels of the Th1-chemokine receptor (CCR5). A 32 basepair deletion in the open reading frame of the gene encoding CCR5 (Δ32) has been shown to be protective against RA in a meta-analysis of RA association studies[78]. We have shown that a variant in the promoter region of *CCR5*, C-1835T is significantly under-transmitted to children with early onset JIA, and with oligoarticular JIA[79]. *CCR5-Δ32 *was also tested in ~700 simplex and multiplex JIA families and found to be under-transmitted to children with early onset JIA. These two variants did not appear to be in LD, and the haplotype that did not contain either of the "protective" variants was significantly over-transmitted. In a study of Norwegian adults with RA and children with JIA, Lindner et al tested *CCR5-Δ32*, and failed to replicate these results[80]. When they repeated the meta-analysis of RA association studies including their results, the negative association between RA and *CCR5-Δ32 *was less pronounced, but still statistically significant (OR 0.8, p < 0.007). When they compared children with JIA and the controls, although there was a trend towards a negative association, it was not statistically significant (OR 0.82, p < 0.15). The authors in that study did not investigate other *CCR5 *variants, including C-1835T.

The gene *PTPN22 *encodes a lymphoid tyrosine phosphatase (LYP) involved in inhibition of T-cell activation. A SNP within the *PTPN22 *gene (C1858T) has been shown to be associated with multiple autoimmune phenotypes including RA, T1DM, and SLE[65,81-83]. To date, there have been four studies of this polymorphism in JIA cohorts [84-86]. The study by Seldin et al did not find an association between 230 Finnish probands with JIA and this *PTPN22 *variant[85]. In contrast, Viken et al reported an association between JIA and *PTPN22 *C1858T using 320 Norwegian cases[86]. Another study by Hinks et al also confirmed an association between *PTPN22 *and JIA using 661 JIA cases from the UK[84]. A fourth study using Czech JIA patients also confirmed the association between the C1858T variant and JIA[87]. A pooled analysis of these four studies confirms that carriage of the T allele increases the susceptibility to JIA, although the magnitude of the association is only modest, with an OR of ~1.3. Thus, *PTPN22 *has been shown to have an association with JIA.

## Strategies to select candidate genes

Selection of candidate genes to be tested for association with JIA might be aided by careful definition of the phenotype, and by testing variants predisposing to monogenic diseases that share phenotypic features with JIA subtypes. For instance, macrophage activation syndrome (MAS), a potentially life-threatening complication, can occur in children with sJIA [88]. MAS is characterized by an overwhelming inflammatory reaction, and is phenotypically similar to hemophagocytic lymphohistiocytosis (HLH). Both familial and acquired forms of HLH are observed. Four autosomal recessive forms of familial HLH have been described, and three of these have been linked to mutations in the genes encoding perforin (*PRF1*), Munc13-14 (*UNC13D*), and syntaxin 11 (*STX11*)[89]. The products encoded by these genes are involved in controlling granule exocytosis in cytotoxic lymphocytes, which are necessary for the physiologic termination of certain immune responses.

Investigations of natural killer (NK) and cytotoxic functions in children with sJIA and MAS have revealed evidence of decreased NK cell activity similar to that seen in familial HLH [90]. In an investigation of NK cell dysfunction, children with sJIA demonstrated significantly decreased NK cell activity compared to pediatric controls, while decreased NK cell activity was rare in children with other JIA subtypes[91]. Grom et al, described three children with sJIA and MAS who demonstrated low levels of perforin expression in all cytotoxic cell populations indistinguishable from that in carriers of perforin-deficient familial HLH[90]. These children did not have *PRF1 *mutations. In a larger study, 62 children with sJIA (some with MAS) were screened for *PRF1 *mutations[92]. Four mutations were identified, out of which, one (Ala91Val) was significantly higher in children who had MAS and sJIA. In a recent study, Zhang et al investigated whether variants in *UNC13D *contribute to MAS in 18 subjects with MAS and sJIA[93]. None of the 18 had mutations in *PRF1*. Two had mutations consistent with a diagnosis of familial HLH. Nine of the remaining 16 had a unique haplotype of 12 SNPs, which was significantly greater than that in controls, (57% vs. 12 %, P < 0.000001), suggesting that this *UNC13D *haplotype is associated with MAS. The frequency of this haplotype in 73 children with sJIA who did not have MAS was not significantly different compared to controls.

Recently, Hazen et al reported on an 8 year old girl with sJIA without clinical evidence of MAS, who carried compound heterozygote mutations in the *UNC13D *gene, and reduced NK cell cytotoxic function[89]. This suggests that some children with sJIA without clinical evidence of MAS have abnormal NK cell function. To estimate the frequency of occult MAS, Behrens et al studied 15 children with sJIA that underwent bone marrow aspirations [94]. Occult MAS was found in 8 subjects, although only 2 had clinical evidence of MAS, suggesting that the immune dysregulation seen in MAS might be integral to the pathogenesis of sJIA. Thus, MAS/HLH could be a part of the spectrum of sJIA, and therefore, it is plausible that genetic variants predisposing to familial HLH might also play a role in susceptibility to MAS, and/or sJIA. Recently, variants in *PRF1, GZMB, UNC13D *and *Rab27a *genes were tested for association with sJIA using 133 cases and 384 controls [95]. None of the variants demonstrated an association with sJIA. Together, these illustrate that careful genotype-phenotype correlations, and extending associations from related phenotypes, will improve the odds of detecting variants predisposing to susceptibility to JIA and its subtypes.

## Genome-wide studies

Another approach to finding genes underlying complex traits is to search the entire genome to find genetic markers that are present more often in affected individuals. Large extended multiplex pedigrees are relatively rare precluding conventional parametric linkage studies, in which parameters such as genetic model, mode of inheritance, penetrance of each genotype etc need to be specified. These parameters are often not available for many complex diseases. An alternative approach is to use non-parametric methods of linkage such as allele-sharing methods in affected sibling pairs. Thompson et al performed a genome wide linkage study using ~400 microsatellite polymorphisms in 121 JIA affected sibling pair families [96]. Although this study confirmed the role of HLA and identified several other regions of interest, the study had limited power and a replication cohort was not available. The heterogeneity of JIA further results in lower power when subjects are stratified by different JIA subtypes. The strongest evidence of linkage to JIA was near the HLA-DRB1 locus. When subjects were stratified by JIA subtype evidence for linkage was found at different chromosomal locations for early onset oligoarticular JIA and polyarticular JIA. This suggests that while some loci such as HLA contribute to JIA susceptibility in general, there are likely other loci that influence the different JIA subtypes. This familiar linkage approach is now being superseded by association studies that involve the entire genome.

An attractive alternative approach to linkage studies is to perform a genome-wide association study (GWAS), which does not require multiplex families, but can be performed with large number of cases and controls. A GWAS examines tens of thousands of genetic variants in a large number of unrelated cases and controls. GWAS have led to the successful identification of several genetic variants that underlie complex autoimmune phenotypes including T1DM, RA, IBD, AS [97-101]. A large collaborative GWAS utilized 3000 shared controls and 2000 cases of 7 different common complex traits which included IBD, RA and T1DM[99]. In all, 17000 individuals were genotyped for >500,000 SNPs. This study confirmed previously reported associations such as *IL23R*, and *NOD2 *for IBD, *HLA-DRB1 *and *PTPN22 *for RA and T1DM as well as several other loci for T1DM[99]. In addition this study identified several new loci that showed strong evidence of association with IBD, RA and T1DM. Some SNPs appear to be associated with more than one autoimmune disease. Other investigators have used GWAS to identify other loci underlying RA such as TRAF1/C5[102]. Interestingly another group using a candidate gene approach also reported an association between RA and TRAF1/C5 locus [103]. GWAS have also been successful in identifying other RA associated loci. Two groups reported associations between individuals with RA and variants located on 6q23, near which are some possible candidate genes[102,104]. Together these studies demonstrate the feasibility of performing GWAS to identify genes underlying predisposition to well defined phenotypes.

To date there are no published studies of GWAS in JIA cohorts although several groups are undertaking such studies. Several GWAS studies are in progress in JIA using high density SNP platforms. Data published in abstract forms suggest that this approach might be successful in identifying JIA associated variants. In a GWAS utilizing individuals with JIA and RA using a SNP platform with 115,075 SNPs, John et al were able to detect known susceptibility loci such as *PTPN22 *and *HLA *[105]. Using a high density SNP GWAS, Thompson et al were able to identify specific polymorphisms potentially involved in JIA at large, as well as other SNPs that appear to influence specific JIA subtypes [106]. Confirmation of initial discoveries from these studies in in independent cohorts is necessary. The consolidation of available cohorts is an active need to improve power, as well as to enable replication studies.

## Genetic variables underlying autoimmunity in general

It has increasingly become clear that autoimmune diseases can cluster both in individuals, as well as in families[107]. We have shown previously, using a case-control design that relatives of patients with JIA have increased prevalence of several autoimmune diseases, especially autoimmune thyroid disease [26]. Others have found increased prevalence of autoimmune thyroid disease or celiac disease in patients with JIA [108-110]. Prevalence of T1DM is also increased in children with JIA[23,111]. Together these studies suggest that clinically distinct autoimmune phenotypes cluster in individuals and families. This supports the hypothesis that common genetic factors might predispose to clinically distinct autoimmune phenotypes.

There are now several concrete examples of genetic variants that influence susceptibility to multiple clinically distinct autoimmune disorders. For instance, a SNP in the *CTLA4 *gene, CT60, has been found to be associated with autoimmune thyroiditis, and T1DM[112]. The same variant has been found to be associated with RA in a meta-analyses [65]. However, this SNP does not appear to play a major role in susceptibility to RF-negative RA or JIA[65,113]. A *PTPN22 *variant has also been associated with multiple autoimmune diseases including RA, SLE, T1DM and JIA. Similarly there are other genetic variants that are associated with multiple autoimmune diseases (Table 5). For instance variants in *TNFA *appear to be associated with RA, JIA, and SLE. Variants in *CCR5 *appear to be protective against both RA and JIA [78,79,114]. Similarly, *IL23R *variants seems to be associated with IBD, Psoriasis and AS, but not with RA [97,101,115-117]. Together, these examples suggest that genetic variants which underlie susceptibility to other autoimmune phenotypes could also potentially be involved in JIA susceptibility. This hypothesis was tested by Thomson et al, who tested *IL2RA *variants associated with both RA and T1DM in children with JIA[118]. *IL2RA *variants were also associated with JIA. These results suggest that investigating genetic variants demonstrating association with multiple autoimmune phenotypes in JIA cohorts might be an attractive approach to find JIA susceptibility genes.

**Table 5:** Examples of genes associated with multiple autoimmune diseases:

Gene	Autoimmune diseases associated
CTLA4	RA, AITD, SLE, T1DM, MS, Celiac disease, autoimmune hepatitis
PTPN22	RA, SLE, T1DM, JIA, Vitiligo, Wegener's, AITD
PDCD1	SLE, RA, T1DM
TNFA	RA, JIA, Crohn's disease
SLC11A1	Crohn's, JIA, RA, T1DM
IL-6	JIA, T1DM, SLE
IL-1A	JIA, RA
MIF	Alopecia areata, JIA, RA, Ulcerative colitis
STAT4	RA, SLE
IL-2RA	T1DM, RA, JIA
CCR5	JIA, RA, MS, T1DM
IL23R	Crohn's disease, Psoriasis, spondyloarthropathy

RA: Rheumatoid arthritis, AITD: autoimmune thyroid disease, SLE: systemic lupus erythematosus, T1DM: type 1 diabetes mellitus, MS: multiple sclerosis, JIA: juvenile idiopathic arthritis,.

In summary, JIA appears to be influenced by genetic factors, both within and outside the HLA region. While many of the HLA associations have been replicated in independent cohorts, only a few of the non-HLA associations have been replicated. The major reason for non-replication appears to be insufficient power. Advances such as genome-wide association studies and collaborative efforts are likely to result in the discovery of new genetic associations, as well as validate (or refute) some of the previously described associations. Finally understanding how a genetic variant associated with JIA and/or other autoimmune diseases influences disease susceptibility is essential to translating findings from the lab to the care of children with JIA.

## Competing interests

The authors declare that they have no competing interests.

## Authors' contributions

SP is the primary author, conceived the article, performed a systematic review of the literature, synthesized the results and wrote the article. DNG is the corresponding author, made substantial contribution to the conception and design of the article, and was involved in revising the manuscript. Both authors read and approved the final manuscript.
